# Identification of small molecules as novel anti-adipogenic compounds based on Connectivity Map

**DOI:** 10.3389/fendo.2022.1017832

**Published:** 2022-12-16

**Authors:** Shuang Zhang, Nicholas Lyons, Marijke Koedam, Jeroen van de Peppel, Johannes P.T.M. van Leeuwen, Bram C. J. van der Eerden

**Affiliations:** ^1^Laboratory for Calcium and Bone Metabolism, Department of Internal Medicine, Erasmus MC, Erasmus University Medical Center, Rotterdam, Netherlands; ^2^Broad Institute of Massachusetts Institute of Technology (MIT) and Harvard, Cambridge, MA, United States

**Keywords:** Connectivity Map, adipogenic differentiation, mesenchymal stromal cell, adipogenesis, microarray

## Abstract

Several physiological and pathological conditions such as aging, obesity, diabetes, anorexia nervosa are associated with increased adipogenesis in the bone marrow. A lack of effective drugs hinder the improved treatment for aberrant accumulation of bone marrow adipocytes. Given the higher costs, longer duration and sometimes lack of efficacy in drug discovery, computational and experimental strategies have been used to identify previously approved drugs for the treatment of diseases, also known as drug repurposing. Here, we describe the method of small molecule-prioritization by employing adipocyte-specific genes using the connectivity map (CMap). We then generated transcriptomic profiles using human mesenchymal stromal cells under adipogenic differentiation with the treatment of prioritized compounds, and identified emetine and kinetin-riboside to have a potent inhibitory effect on adipogenesis. Overall, we demonstrated a proof-of-concept method to identify repurposable drugs capable of inhibiting adipogenesis, using the Connectivity Map.

## Introduction

Bone marrow adipocytes account for approximately 70% of the marrow volume representing a major population of marrow cells (1, 2). Bone marrow adipose tissue (BMAT) possesses a unilocular morphology and are considered to have originated from bone marrow-derived mesenchymal stromal cells (BMSCs). The cell morphology, lineage origin and its response in various physiological conditions make BMAT a unique population, which is anatomically and functionally distinct from white, brown and beige adipocytes (3, 4). This is supported by differential gene expression profiles, since bone marrow adipocytes (BMAds) express reduced lipid and increased cholesterol metabolism markers versus subcutaneous adipocytes (5). BMAT also differs from brown adipose tissue (BAT) since during aging, BAT markers are decreased in BMAds (6). BMAT is a critical regulator of the bone marrow environment through at least two distinct mechanisms: 1) by release of cytokines resulting in paracrine effects, and 2) by direct interaction with other types of cells (4).

Population studies have suggested an inverse relationship between BMAT and bone mineral density in aged humans as well as even in young individuals (4, 7). Lineage tracing and single cell RNA-seq experiments demonstrated adipogenesis-primed MSCs expressed high levels of leptin receptor (LEPR) and C-X-C motif chemokine ligand 12 (CXCL12), while these markers decreased in osteogenesis-primed MSC (8, 9). Studies in mice showed that parathyroid hormone treatment increased bone mass while it reduced marrow adipocyte number (10–13). These studies combined, together with a recent bone marrow-based single cell RNA-seq study indicating bifurcation of osteogenic and adipogenic differentiation from BMSCs (14), implicate a reciprocal relationship between the two differentiation lineages. There is preference for osteogenesis at the expense of adipogenesis and this changes towards a preferential adipogenic differentiation in several physiological and pathological conditions including aging, obesity, diabetes, anorexia nervosa (15), leading to bone fragility and potential fractures (16–18). Together with the pathological consequences mentioned above, such disruptions can significantly reduce the patients’ quality of life and therefore effective ways of controlling dysregulated adipogenesis could be important.

Our previous study using Connectivity Map (CMap) identified parbendazole as a potent compound to promote osteogenesis (19). CMap is a library of over 1.5M gene expression profiles of about 5000 small molecules and 3000 genetic reagents tested in multiple cell types allowing genome-wide investigation of connections between pharmacological perturbations and queries of up- and down-regulated gene sets from experimental conditions (20, 21). It is achieved by holistically quantifying the relationships between user-selected sets of differentially expressed (DE) genes and a compendium of 1.3 million of L1000, a high-throughput gene expression profile, covering 42 thousand perturbagens for a total of 473,647 signatures (21). This query creates a ranked list of compounds with biological signatures highly correlated to the cellular expression patterns of an experimental condition at high levels of resolution and specificity. This facilitates screening of compounds for novel disease treatments and targeting biological processes, and help understanding molecular machineries in complex biological pathways. To date, the power of CMap has been broadly demonstrated in novel drug identification and combination therapies in various diseases and biological events (22–26).

In this study, we applied a transcriptomics approach to predict compound-induced perturbations of BMSC-derived adipogenesis using CMap, and further conducted a transcriptomic screen of BMSCs using the prioritized compounds. Finally, we treated BMSCs with 3 selected compounds to validate the CMap predictions. This study provides new insights into the discovery of small molecules to control adipogenic differentiation of BMSCs, and paves the way for drug repurposing in maintaining the balance between adipocytes and osteoblasts.

## Materials and methods

### Cell culture and differentiation

Human bone marrow-derived mesenchymal stromal cells (BMSCs) were purchased from Lonza (Geleen, the Netherlands) and maintained as previously described (23, 27, 28). Two days after seeding, BMSCs were initiated to adipogenic differentiation using 100 nM dexamethasone, 60 μM indomethacin, and 0.5 mM 3-isobutyl-1-methylxanthine. BMSCs at passage 7 were used in the experiments. Culture media was refreshed every 3 or 4 days with various concentrations of compounds during the courses of inductions. These cultures are called adipogenic-BMSCs throughout the remainder of the study.

All tested compounds in this study were either provided by the CMap research group at the Broad Institute or purchased from Sigma (Sigma-Aldrich, St. Louis, MO, USA).

### Oil red O staining

Adipogenic-BMSCs were fixed with 10% formalin, and subsequently incubated with Oil red O solution (Sigma-Aldrich, St. Louis, MO, USA) for 30 min at room temperature (RT) as described (28).To determine cell number, DAPI was used to stain for nuclei. Images (5 per well) were taken using a Zeiss Axiovert 200MOT microscope (Zeiss, Sliedrecht, the Netherlands) and DAPI positive staining was counted using Fiji software. Cell number was calculated from the average of 5 images in each well. Lipid droplets were extracted with isopropanol and absorbance was subsequently measured at 490 nm. Normalized absorbance was calculated as the raw absorbance divided by cell numbers.

### Cell viability assay

Cell viability was determined using Cell Counting Kit-8 (CCK-8) assays. Adipogenic-BMSCs were incubated with CCK-8 reagent (Sigma-Aldrich, St. Louis, MO, USA) for 2 hours in an incubator according to the manufacturer’s manual. The conversion of the tetrazolium salt WST-8 to formazan was measured at 450 nm.

### RNA isolation and quantification of mRNA expression

RNA isolation and cDNA synthesis were performed as described (28). Oligonucleotide primer pairs for qPCR were designed to be either on exon boundaries or spanning at least one intron (**Table S1**). qPCR was performed using the GoTaq qPCR Master Mix system (Promega, Madison, WI, USA). Gene expression was normalized to the expression of *36B4* using the equation 2^^- (Ct gene of interest – Ct housekeeping gene)^.

### Illumina gene chip-based gene expression profiing and data analysis

Illumina Human HT-12v3 BeadChip arrays were performed as described (29). RNA was isolated from three biological replicates of adipogenic-BMSCs at day 4 and day 13 as described (28). RNA concentration and size distribution profiles were assessed on a 2100 Bioanalyzer (Agilent Technologies B.V., The Netherlands). RNA was amplified using Illumina TotalPrep RNA Amplification Kit according to manufacturer’s instructions (ThermoFisher Scientific, Massachusetts, USA). A total of 750 ng cRNA was hybridized using the standard protocol from Illumina. Data was analyzed after background subtraction using GenomeStudio (V2010.1, Illumina), and processed with R2.10.1 lumi-package in R (30). Differentially expressed (DE) probes (adjusted *p*-value < 0.001) were identified when comparing to undifferentiated BMSCs using Iimma package from Bioconductor (31).

### CMap query

A connectivity mapping approach was used to prioritize small molecules as previously described (19, 23). The similarities between gene expression profiles from microarray analyses and reference signatures in CMap are computed (21). Top 100 up- and down-regulated probes based on the log_2_-fold change were submitted to the query version 1.0 (Dataset S1, https://clue.io/query). The summary score is a perturbagen-centric measure of connectivity that summarizes the results observed in individual cell types, while the connectivity score is a quality control measurement comparing an observed enrichment score from adipogenic gene set to reference gene sets, ranging from -100 to 100. A positive score suggests the facilitated effect on adipogenesis, while a negative score suggests an inhibitory effect on adipogenesis. The magnitude of the score corresponds to the magnitude of similarity or dissimilarity, and the tops and/or bottoms of these connectivity lists can be thought of as hypotheses about the about the relationship of the perturbagen to the query. For further details on these scores, see https://clue.io/connectopedia/.

### Cell lysate preparations, expression profiling and data analyses

BMSCs cultured in 96-well plates were treated for 24 h with the compounds following 3 days of adipogenic induction. Compounds used in the screening process were provided by the CMap research group at the Broad Institute in heat-sealed 384- well plate. The concentrations of compounds (3 replicates per concentration per compound) used in adipogenesis cultures were determined based on the data in the L1000 database (21). The vehicle of the compounds, dimethylsulfoxide (DMSO), served as negative control and was added in the same treatment plate. Cell lysate was prepared by using 35 μl of TCL lysis buffer (QIAGEN, Hilden, Germany). The plates were sealed with adhesive film and incubated for 30 minutes at RT prior to storage at -80°C. Compound-induced gene expression profiles were generated using the L1000 platform (21). Raw fluorescent intensity data were detected by Luminex scanners and further deconvoluted to generate expression profiles of 978 landmark genes. Additional values of 12,328 genes not directly measured were inferred based on normalized values of the landmark genes. Compound signatures were represented using robust Z-scores (RZS), which reflect the degree to which a gene was up or down-regulated in a given sample relative to all other samples on the same plate (21). A compound - gene perturbation was classified as DE genes between control and treatment if the absolute difference in median RZS was ≥ 2. Gene set enrichment analysis (GSEA) was performed on the robust Z-score using HALLMARK gene sets in the Molecular Signatures Database (MSigDB) (32).

### Statistical analysis

Data were expressed as means ± SEM of representative experiments. All experiments were performed at least 2 times. Statistical analysis was performed using GraphPad Prism 9 or R package. Significance was calculated using Student’s *t*-test, one-(two) way ANOVA following post-hoc test. *P* value less than 0.05 was considered significant.

### Data availability

All of the microarray data and CMap data that are at the basis of this study have been deposited in Gene Expression Omnibus (GEO) with the accession numbers GSE80614 and GSE70138, respectively.

## Results

**Figure 1:**
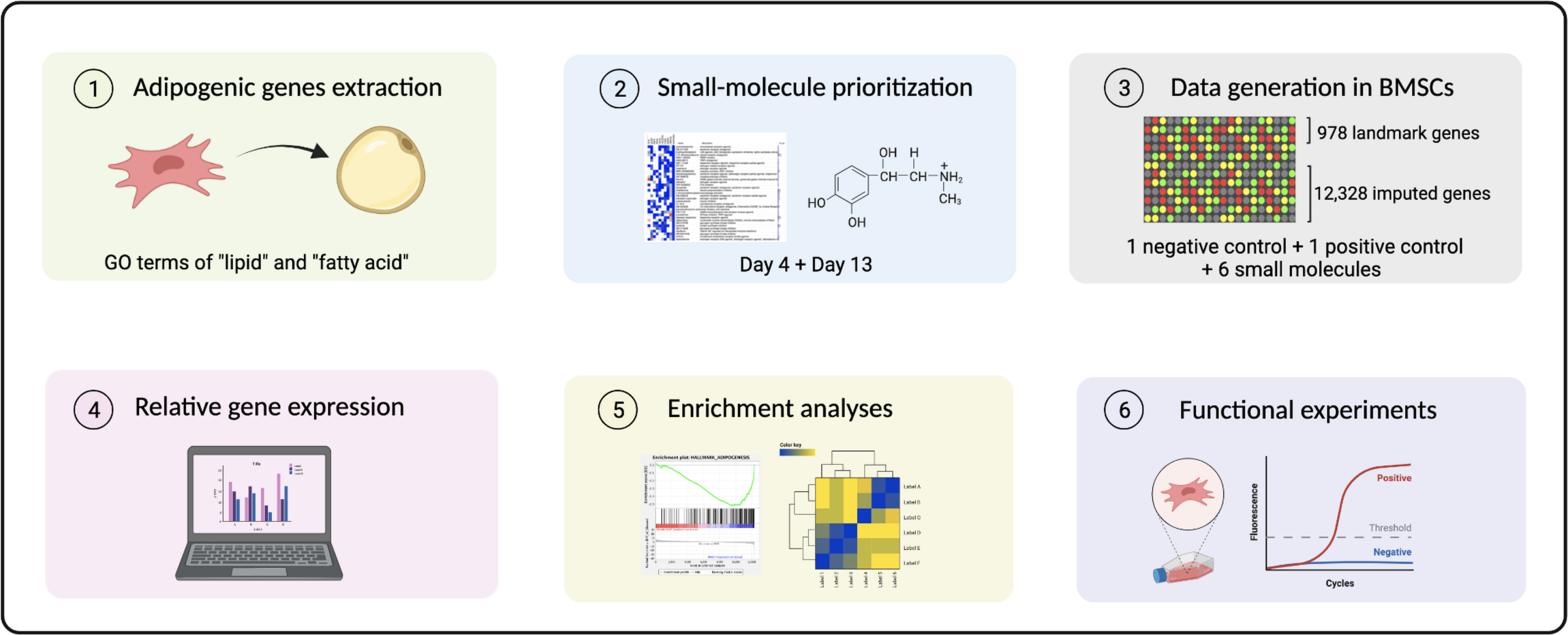
A schematic overview of analytical and experimental pipeline. ^1^Differentially expressed (DE) probes at day 4 and day 13 annotated by GO terms “lipid” and “fatty acid” were extracted. ^2^ Small molecules were prioritized for further screening based on a connectivity-mapping strategy. ^3^ Transcriptomic profiles were generated in BMSCs using treatment with selected compounds using the L1000 platform. ^4^ Relative gene expression indicated using a robust Z-score, ^5-6^ together with enrichment analyses provided evidence for functional experiments.

An overview of our experimental pipeline is depicted in **Figure 1**. Following the upload of our microarray-based top 100 up- and downregulated gene lists at day 4 and 13 of adipogenic differentiation, small molecules were prioritized for screening based on the rank of the order in the query. We applied a transcriptomic screen to assess compound-induced perturbations using adipogenic-BMSCs, a primary human multipotent stromal cell type with the capacity to differentiate into adipocytes, which is far more relevant to our research question than cancer cell lines historically employed in the LINCs datasets. This *in silico* approach was conducted using the L1000 assay pipeline, which allows for comparing results to the query. Following enrichment analyses, functional experiments were performed to confirm the inhibitory effect of compounds discovered in the query.

### Identification of up- and down-regulated adipogenic differentiation-specific genes

To identify genes that were differentially regulated during adipogenesis, we differentiated BMSCs with adipogenic induction medium for 4 days and 13 days representing the early and late adipocyte differentiation phase, respectively. Cells at day 0, 4 and 13 were collected for microarray-based gene expression analyses. DE probes at day 4 and day 13 were identified by comparing these with the non-induced cells at day 0. Transcriptomic snapshots cannot distinguish causal genes of the adipogenic-BMSCs phenotype from the effect genes, which are in physiological feedback loops and may contribute to a bias over a single representative of such a cluster (33). Therefore, we decided to use a reduced gene set by extracting the top 100 up or down-regulated DE probes, which are more specific to the unique characteristics of adipocytes.

**Figure 2:**
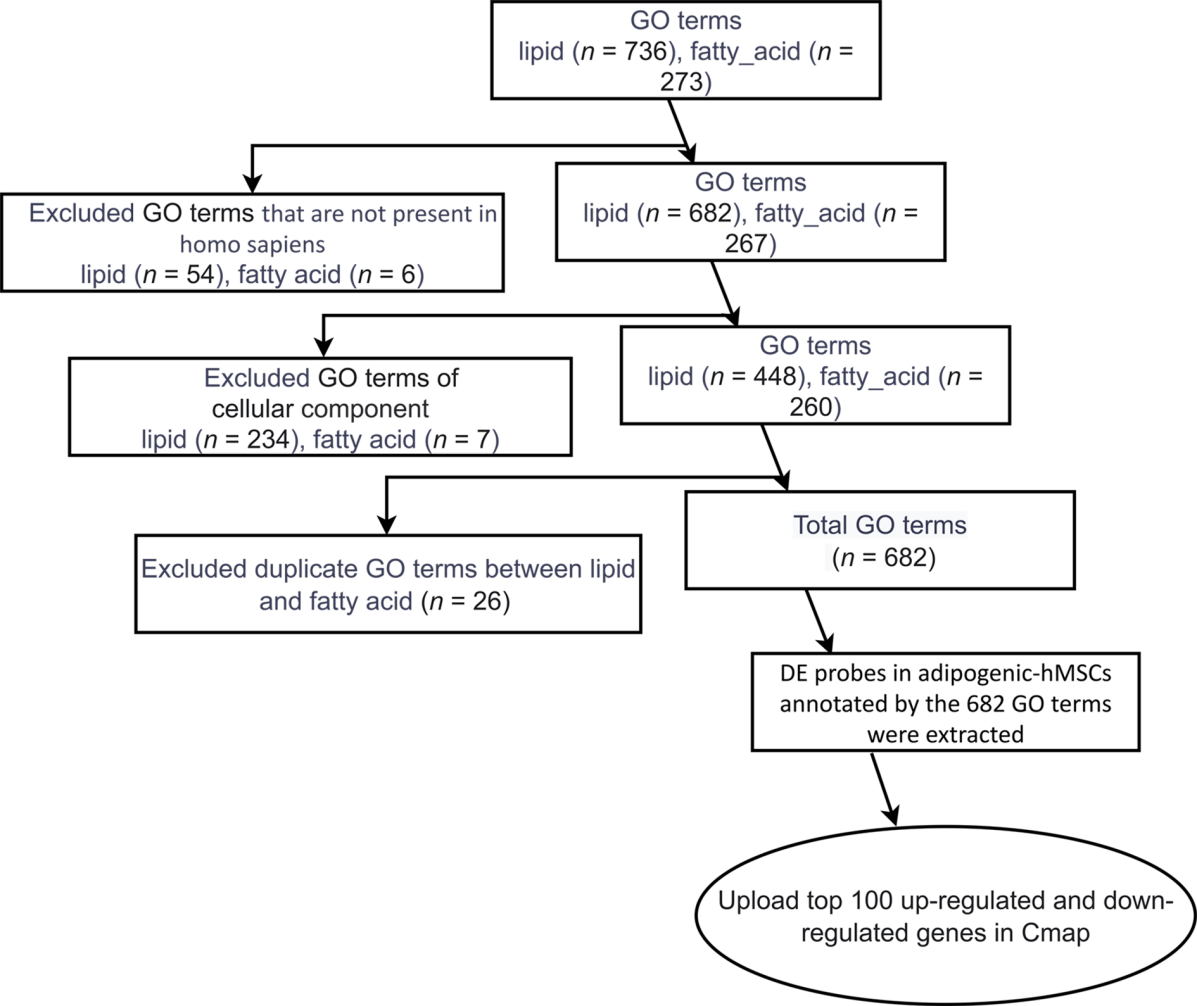
Identification of lipid- and fatty acid-associated genes. A flow chart describing the steps to filter Gene Ontology (GO) terms related to lipid and fatty acid, resulting in the final gene lists used for CMap analysis.

To identify those unique genes, we utilized Gene Ontology (GO) annotations because of its query specificity and ability to distinctively annotate molecular functions, biological processes and cellular components. We began with extracting GO terms related to “lipid” and “fatty_acid”, resulting in 736 and 273 terms respectively (**Figure 2**). Terms that were not present in homo sapiens (54 in “lipid” and 6 in “fatty_acid”) were excluded. Given our aim to identify compounds that can inhibit molecular pathways or cellular processes during adipogenic differentiation, instead of the formation of certain cellular structures, such as mitochondria or ribosome-enriched in adipocytes, we focused on Molecular Functions and Biological Processes and terms under “cellular component” (234 in “lipid” and 7 in “fatty_acid”) were removed. By further eliminating 26 overlapping GO terms between the “lipid” and “fatty_acid” filters, a total of 682 GO terms specific to molecular functions and cellular events of human adipogenic differentiation were identified. Up- and down-DE probes at day 4 and day 13 annotated by the 682 GO terms were extracted, and the top 100 up- and down-DE probes at these two time points were used for the adipogenic differentiation-specific queries in CMap analyses.

### Small molecule-prioritization from CMap query

**Figure 3:**
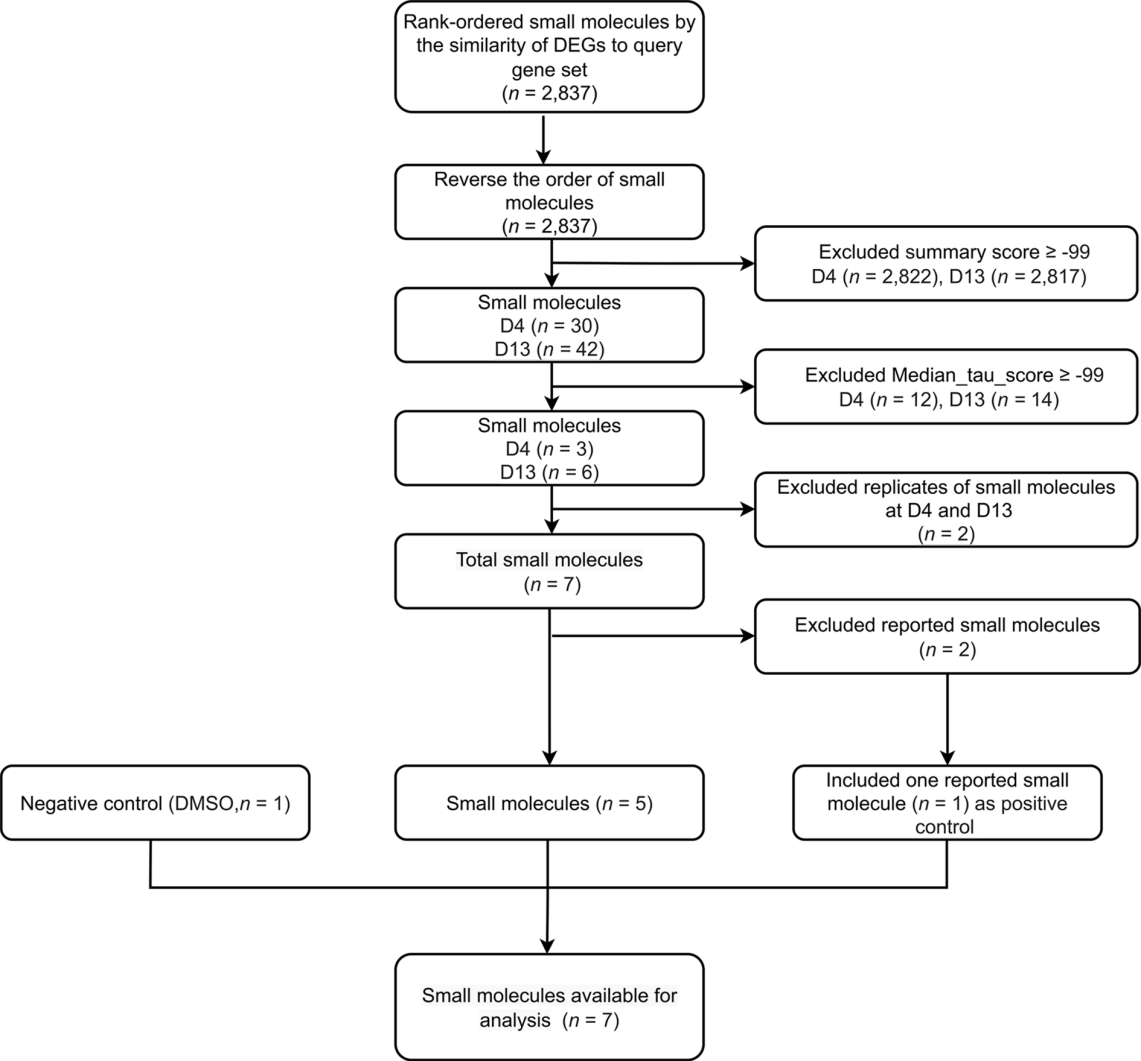
Step-by-step illustrated procedure for small molecule-prioritization. A flow chart outlining the selection criteria applied to select small molecules, using CMap.

**Figure 4:**
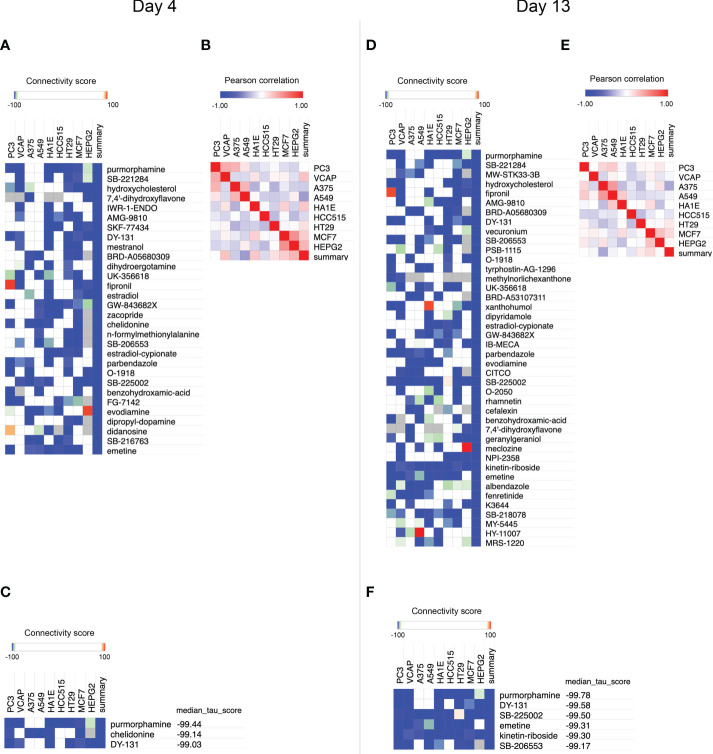
Small molecule-prioritization from CMap query **(A)** Heatmap showing compounds with a summary score less than -99 using differentially expressed (DE) probes of microarray data derived from adipogenic-BMSCs at day 4. **(B)** Expression similarities among different cell lines are indicated at day 4. **(C)** Compounds with a connectivity score and a summary score less than -99 were identified using microarray data at day 4. **(D)** Heatmap showing compounds with a summary score less than -99 using DE probes of microarray data derived from adipogenic-BMSCs at day 13. **(E)** Expression similarities among different cell lines were indicated at day 13. **(F)** Compounds with a connectivity score and a summary score less than -99 were identified using microarray data at day 13. Compounds were sorted by ascending order of median connectivity (tau) score in **(C)** and **(F)**.

To identify compounds that inhibit adipogenesis, the top 100 up- and down-gene lists at day 4 and day 13 were submitted to CMap as two separate queries. For each query, the entire small molecule library, containing 2,837 chemicals, was ranked based on summary scores of each molecule representing signatures from total 9 cell lines (**Figures 3**, **S1**, **S2**). Summary scores were computed using a perturbagen-centric measure of connectivity that summarizes the results observed in individual cell types (20). The positive scores indicate similarity between input gene sets from the list of DE probes and gene sets associated with each molecule, in this case meaning pro-adipogenic, while negative scores indicate an opposite association implicating it to be anti-adipogenic. Therefore, we reversed the list and only focused on the negative scores. Small molecules of which the summary scores are greater or equal to -99 were removed to select the most anti-adipogenic compounds (**Figure 3**). At day 4, 30 compounds were found (**Figure 4A**), whereas 42 compounds were found at day 13 (**Figure 4D**). Pearson correlation analyses of gene set dissimilarities among all 9 cancer cell lines in the CMap database showed that the list of anti-adipogenic candidates had no clear preferential effect on a particular type of cells in both queries, indicating the summary score was equally influenced by all 9 cell lines (**Figures 4B, E**). To further enrich the lists, we applied the connectivity score comparing an observed enrichment score from adipogenic gene set to reference gene sets based on L1000 platform, ranging from -100 to 100. A connectivity score less than -99 means only less than 1% of reference gene sets showed stronger dissimilarity than a given molecule in the query lists. Compounds with median connectivity scores greater or equal to -99 were deleted, resulting in 3 compounds and 6 compounds at day 4 and day 13 queries, respectively (**Figures 4C, F**). Since purmorphamine and DY-131 were present in both queries, we identified 7 potential anti-adipogenic candidates in total. Purmorphamine and chelidonine had been tested for their anti-adipogenic effects in BMSCs (34). Since purmorphamine ranked top 1 in our CMap analyses, demonstrating the validity and strength of our approach, we therefore included purmorphamine as a positive control in the downstream functional analyses. In total, we generated a list of 6 anti-adipogenic candidate from a systematic CMap analysis (**Table 1**), and 5 of them were eventually used in further experiments. Apart from kinetine-riboside, all the compounds are either in the preclinical phase or phase 2 of clinical trials.

**Table 1:** Candidate compounds computed from CMap potentially targeting adipogenic differentiation.

Name	Median_tau_score^1^	Summary score^2^	Description	Target	Clinical phase
purmorphamine	-99.78	-99.93	smoothened receptor agonist	SMO, DHH, IHH, PTCH1	Preclinical
DY-131	-99.58	-99.82	estrogen-related receptor agonist	ESR2, ESRRB, ESRRG	Preclinical
SB-225002	-99.50	-99.51	CC chemokine receptor antagonist, Chemokine CXCR2 antagonists	CXCR2	Preclinical
emetine	-99.31	-99.19	protein synthesis inhibitor	RPS2	Approved
kinetin-riboside	-99.30	-99.19	apoptosis inducer		
SB-206553	-99.17	-99.79	serotonin receptor antagonist, serotonin receptor partial agonist	HTR2B, HTR2C, HTR1A, HTR2A	Preclinical

^1^ Median tau score: Ranging from 100 to −100, correlated to reference gene sets, generated by previously well annotated molecules in the L1000 platform. ^2^ Summary score: A perturbagen-centric measure of connectivity that summarizes the results observed in individual cell types. ^3^Clinical phase: indicates the trial phase of the compound.

### Prioritized compounds reflect different transcriptome responses from vehicle compound

**Figure 5:**
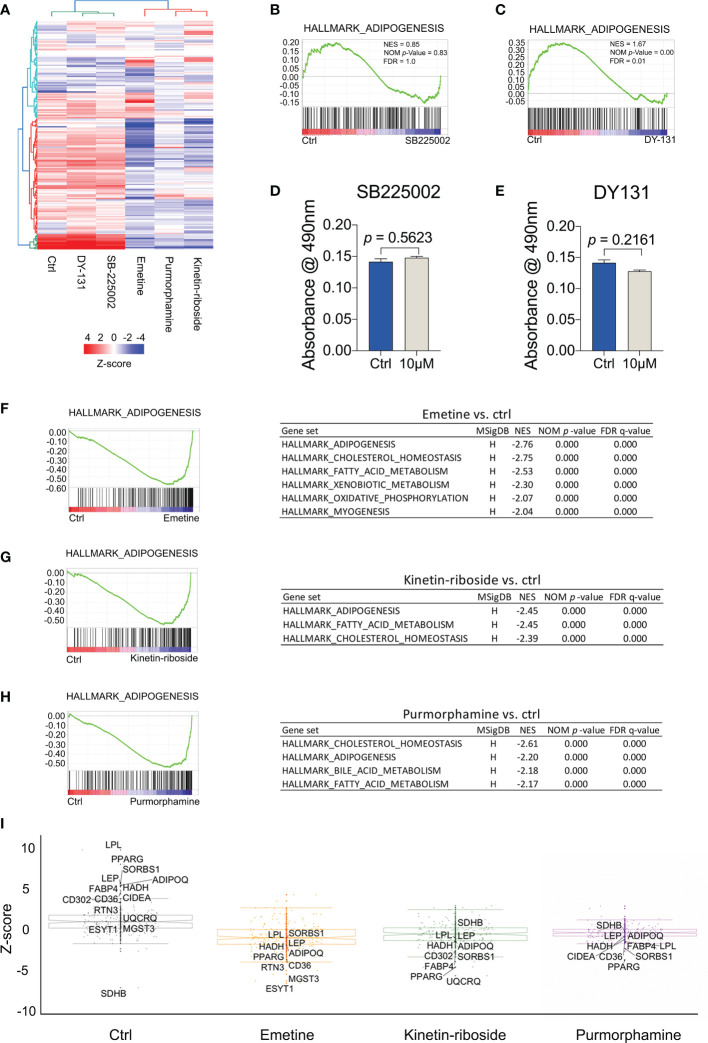
Transcriptomic signatures of BMSCs treated with prioritized compounds. **(A)** Heat map showing the expression pattern of emetine, kinetin-riboside and purmorphamine constructed with adipogenesis-related genes identified in the HALLMARK_ADIPOGENESIS gene set from The Molecular Signatures Database (MSigDB). **(B, C)** Gene set enrichment analyses (GSEA) of adipogenesis pathway following the treatment of SB225002 **(B)**, DY-131 **(C)**. **(D, E)** quantitative data of Oil Red O staining. Oil Red O staining was performed after 14 days of adipogenic differentiation from BMSCs following SB225002 **(D)** or DY-131 **(E)** treatment. **(F–H)** GSEA of decreased adipogenesis pathway following the treatment with emetine **(F)**, kinetin-riboside **(G)** and purmorphamine **(H)**. Gene sets with a NES less than -2 were shown in tables. **(I)** Box plots showing DE genes using robust Z-score (RZS). Compounds signatures were quantified by transforming normalized gene expression to RZS. The top 10 differentially expressed **(D, E)** genes with an absolute difference in RZS ≥ 2 (compound vs ctrl) following treatment with emetine, kinetin-riboside and purmorphamine were labeled with gene names. All gene names shown in the compound graphs were included in the control graph. Normalized enrichment score (NES), NOM p-value (normalized *p*-value), false discovery rate (FDR).

Given that the compound prioritization was performed using cancer-related cell lines, we decided to evaluate their responses in BMSCs as they comprise the stem cells of bone marrow adipocytes. To this end we analyzed the gene expression profiles of adipogenic-BMSC treated with the test compounds or the negative and positive controls. A heat map was constructed with adipogenesis-related genes identified in the HALLMARK_ADIPOGENESIS gene set from the Molecular Signatures Database (MSigDB) (**Figure 5A**). Our analyses revealed two distinct groups among the 5 compounds, of which DY-131 and SB-225002 were clustered together with the control condition, while emetine and kinetin-riboside showed an opposite expression profile compared to the control group (**Figure 5A**), which was similar to the previously characterized adipocyte inhibitor purmorphamine. GSEA analyses indicated that adipogenesis genes were either not or positively enriched following treatment with SB-225002 or DY-131 in adipogenic-BMSCs, respectively (**Figures 5B, C**). These assumptions were further confirmed by Oil Red O assays showing no effect of SB-225002 or DY-131 on adipogenesis (**Figures 5D, E**). Therefore, we anticipated that emetine and kinetin-riboside are both potential compounds suppressing adipogenesis.

Indeed, GSEA analyses for the emetine, kinetin-riboside and purmorphamine treatments demonstrated enrichments for adipogenesis, cholesterol homeostasis and fatty acid metabolism opposite to the control condition (**Figures 5F–H**). Importantly, we found that adipogenesis marker genes such as *PPARG*, *LPL*, *ADIPOQ* and *LEP* were significantly downregulated in the presence of emetine, kinetin-riboside or purmorphamine in adipogenic-BMSCs (absolute difference in RZS ≥ 2, **Figure 5I**). Taken together, these findings suggest that these 3 compounds induce negative perturbations compared to the control group, suggesting anti-adipogenic effects.

### Anti-adipogenic effects using emetine, kinetin-riboside and purmorphamine

To verify if emetine, kinetin-riboside and purmorphamine have anti-adipogenic effects, *in vitro* BMSC adipogenesis assays were performed. Adipogenic-BMSCs were cultured for 14 days in the presence of each compound at two different concentrations, or with the vehicle control and next examined by Oil-red O analyses. For each compound, the higher concentration was corresponding to the concentration used in CMap database or published data (35), being 0.1 μM for emetine and 10 μM for kinetin-riboside and purmorphamine, where the lower concentration was one-third of the higher concentration.

**Figure 6:**
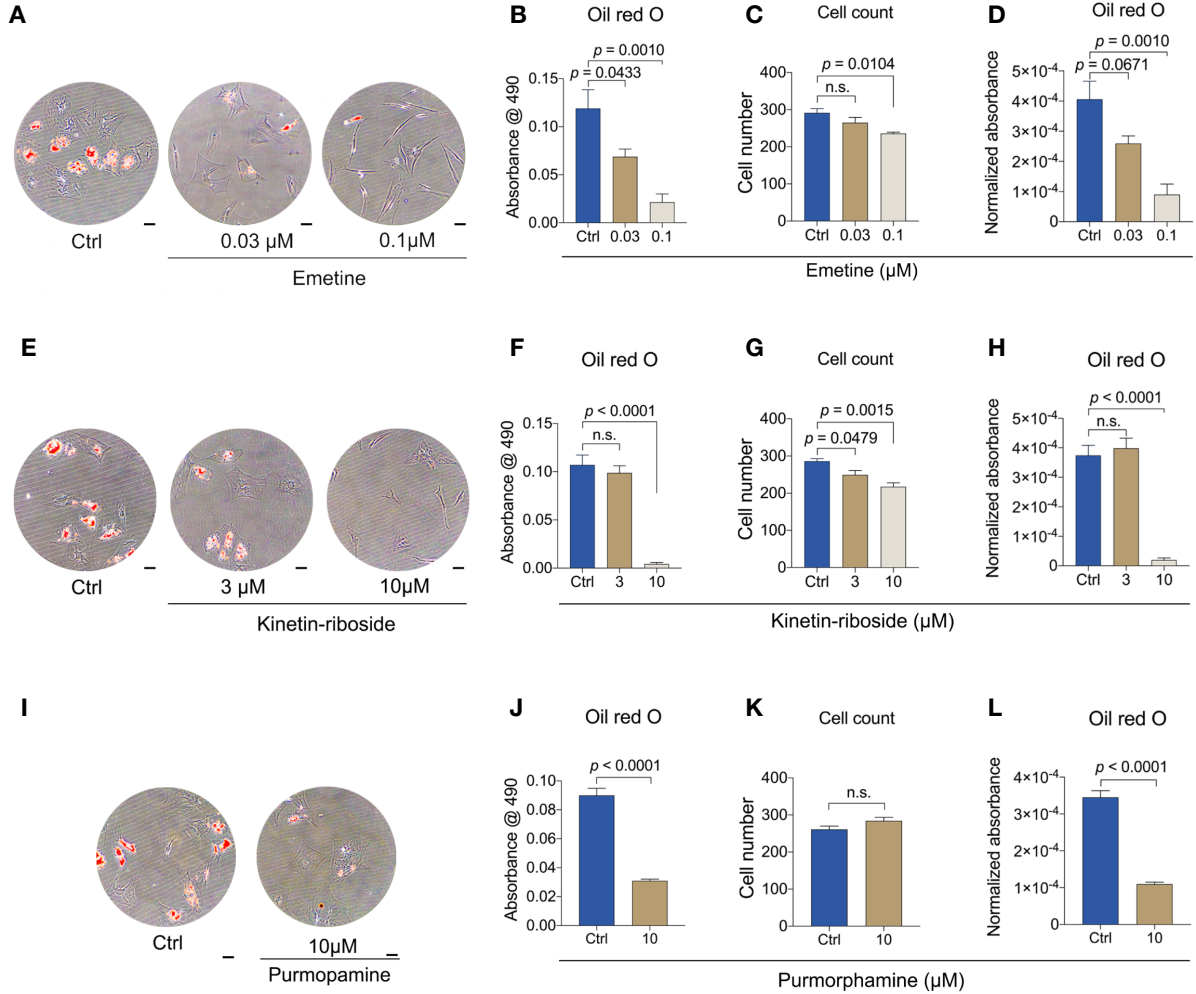
Emetin, kinetin-riboside and purmorphamine are anti-adipogenic. **(A-D)** Representative images **(A)** and quantitative data **(B-D)** of Oil Red O staining. Oil Red O staining was performed after 14 days of adipogenic differentiation from BMSCs following emetine treatment **(B)**. Normalized absorbance of Oil Red O **(D)** was adjusted by cell number **(C)**. **(E-H)** Representative images **(E)** and quantitative data **(F-H)** of Oil Red O staining as described in **(A-D)** following treatment of kinetin-riboside using adipogenic-BMSCs. **(I-L)** Representative images **(I)** and quantitative data **(J-L)** of Oil Red O staining as described in **(A-D)** following treatment of purmorphamine. Scale bars in A, E and I: 50 μm.

The presence of emetine at either 0.03 μM or 0.1 μM significantly reduced Oil red O staining (**Figures 6A, B**). Total Oil red O absorbances decreased with increasing emetine concentrations in a dose-dependent manner (**Figure 6B**). In agreement with the reduction in cell number, cell viability indicated by CCK-8 experiment was decreased, suggesting a potential negative effect on adipogenic progenitor proliferation or survival of adipocytes (**Figures 6C** and **S3**). A similar dose-dependent response was also shown for normalized Oil red O absorbance (**Figure 6D**).

Kinetin-riboside also showed a clear Oil red O reduction at 10 μM, but there was no distinguishable difference between the 3 μM and control group (**Figures 6E, F**). Total cell number as well as the cell viability showed decreases with both concentrations (**Figures 6G** and **S3**). Cell number-normalized Oil red O also showed only for 10 μM a strong decrease (**Figure 6H**).

The positive control purmorphamine (10 μM) also caused a decrease in Oil red O (**Figure 6I**) both uncorrected and corrected for total cell number (**Figures 6J, L**). Purmorphamine at this concentration did not change cell number and cell viability (**Figures 6K** and **S3**).

**Figure 7:**
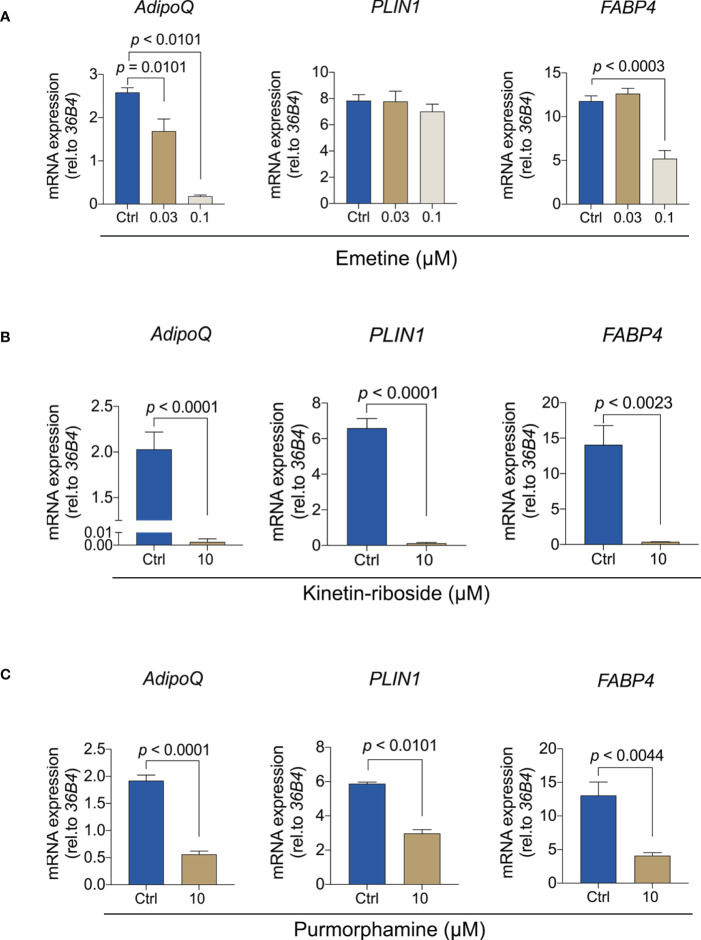
Emetine, kinetin-riboside and purmorphamine inhibit the expression of adipogenesis genes. **(A-C)** The expression of *ADIPOQ*, *PLIN1* and *FABP4* were evaluated after 7 days of adipogenic induction from BMSCs following treatment with emetine **(A)**, kinetin-riboside **(B)** and purmorphamine **(C)**.

To provide more insights into the molecular effect of these compounds, we analyzed the expression of adipogenesis genes (**Figure 7**). Emetine showed a dose-dependent inhibition of the expression of *ADIPOQ* but that was not observed in *PLIN1*, consistent with the transcriptomic analyses. Only high doses of emetine led to *FABP4* downregulation (**Figure 7A**). Since lower doses of kinetin-riboside did not lead to the reduction of Oil red O absorbance, we only evaluated the expression of adipogenic-specific genes using the high dose of kinetin-riboside. *ADIPOQ*, *PLIN1* and *FABP4* were significant decreased in the presence of 10 μM kinetin-riboside (**Figure 7B**). Similar effects were observed following the treatment with the positive control purmorphamine (**Figure 7C**). Collectively, these data indicate that emetine, kinetine-riboside and purmorphamine all suppress the differentiation of adipogenic BMSCs.

### Effects of emetine, kinetin-riboside and purmorphamine on different stages of adipogenesis

**Figure 8:**
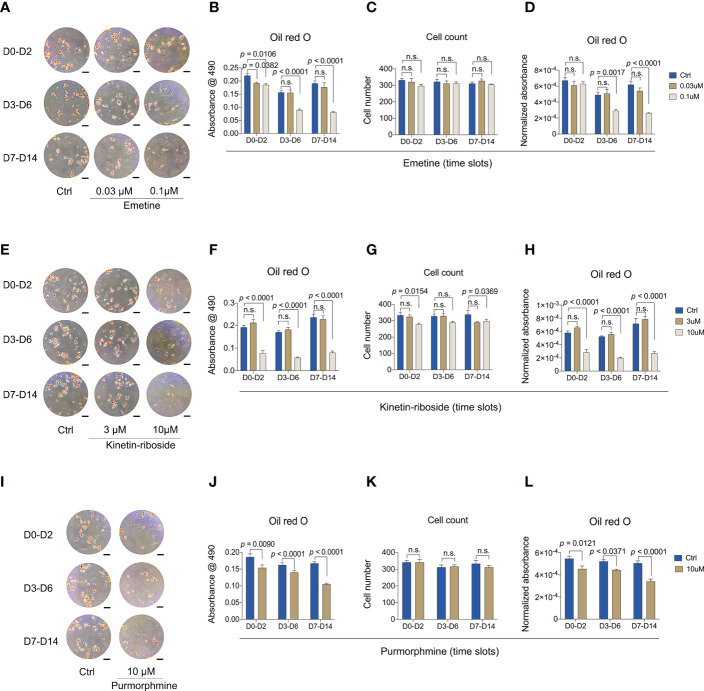
Anti-adipogenic effects of emetine, kinetin-riboside and purmorphamine are independent of treatment window. **(A-D)** Representative images **(A)** and quantitative data **(B-D)** of Oil Red O staining. Oil red O staining was performed after 14 days adipogenic differentiation from BMSCs following emetine treatment at indicated time slots. **(E-H)** Representative images **(E)** and quantitative data **(F-H)** of Oil Red O staining. Oil Red O staining was evaluated after 14 days adipogenic induction following treatment with kinetin-riboside at indicated time slots. **(I-L)** Representative images **(I)** and quantitative data **(J-L)** of Oil Red O staining. Cells were cultured in adipogenic induction medium following treatment with purmorphamine at indicated time slots. Normalized absorbance of Oil Red O **(D, H, L)** was adjusted by cell number **(C, G, K)**. Scale bars in A, E and I: 100 μm.

After verifying their bona fide anti-adipogenic effects, we wanted to understand whether such effects were related to different stages of adipogenic differentiation. We therefore divided the 14-day culture time window into three stages, early stage (from day 0 to day 2), mid stage (from day 3 to day 6), and late stage (from day 7 to day 14). Compounds at two concentrations were added only during each of the stages, and cells were collected at day 14 for Oil red O staining and quantification (**Figure 8**).

Addition of emetine at either concentration during early stage adipogenesis resulted in a mildly reduced Oil red O staining (**Figures 8A, B**). Significant reductions in Oil red O staining were shown when 0.1 μM emetine was added at mid and late stages, indicating emetine might preferentially target mid and late stages of adipogenic differentiation. Short-term treatment with 0.03 μM emetine was not effective suggesting emetine might need longer incubation times to demonstrate a full anti-adipogenic effect at this concentration (**Figures 8A, B, D**). Compared to vehicle treatment, mid and late stages incubation of emetine led to 40% and 58% decrease, respectively, which were not as strong as the 14-day incubation (78% decrease vs. vehicle) as shown in **Figure 6D**. It is worth noting that stage-wise incubation of emetine did not lead to any effect on cell count, indicating its negative effect might be dependent on incubation duration (**Figure 8C**).

Kinetin-riboside, on the other hand, showed clear Oil red O staining reduction when it was added during any of the three stages with the 10 μM dose (**Figures 8E, F**). However, the degree of reduction in total and normalized Oil red O absorbance here was less dramatic than that of the 14-day incubation (**Figures 8F, H**), with 51%,62% and 62% decrease in the early, mid and late stages, respectively, while decreasing by 95% in continues treatment of kinetin-riboside compared with controls. The reduced cell count was still obvious when using the 10 μM dose, but it was also less severe compared to the 14-day incubation, while the negative effect at 3 μM was not observed here (**Figure 8G**).

Purmorphamine also produced an anti-adipogenic effect as compared with kinetin-riboside in reducing Oil red O staining at all three stages (**Figures 8I–J**). Similarly, the degree of total and normalized Oil red O absorbance reduction here was smaller than that in the 14-day incubation (**Figures 8K, L**), showing as 17%, 15%, 33% decrease in the early, mid, later incubation stages, and 68% decrease for continues incubation of purmorphamine compared with controls. Together, these findings suggest that the anti-adipogenic response of BMSCs is primarily driven by the duration of the treatment using any of the 3 compounds.

## Discussion

The present study conducted a novel strategy to identify two novel compounds, emetine and kinetin-riboside, that inhibit adipogenesis based on a connectivity-mapping approach. We extracted the genes driving the GO terms related to lipid and fatty acid and assessed the expression (direction and magnitude) of these genes in adipogenic-BMSCs. Next, the Top 100 genes with most strongly altered expression, either up or down, were selected and used for CMap analyses. Following CMap database scrutiny small molecules tending to reverse the response of adipogenic differentiation were prioritized based on summary and connectivity scores. In this way we identified purmorphamine, a hedgehog agonist directly stimulating smoothened (36). The anti-adipogenic effects of hedgehog signaling and of purmorphamine have already been reported (34, 37, 38). This supports the strength of our current approach to identify compounds that inhibit adipogenesis. Of all 6 compounds prioritized in this way *in silico*, we selected further applying a transcriptomic screen in BMSCs following adipogenic differentiation. Compound-induced gene expression changes observed in BMSCs were strongly enriched for genes linked to adipogenesis. Finally, we demonstrated that two compounds predicted by CMap, emetine and kinetin-riboside, are able to inhibit adipogenic differentiation of BMSCs.

### CMap as a novel tool to discover and prioritize small molecules related to adipogenesis

The use of CMap has already led to the identification of anti-adipogenic molecules for obesity therapy (39, 40). However, to the best of our knowledge, our work represents the first study using this novel method for small-molecule prioritization, and further scale down the screening by analyses of transcriptomic signatures of BMSCs to support the discovery of novel biology and therapeutic targets against adipogenesis. The extraction of genes from GO terms related to ‘lipid’ and ‘fatty acid’ are triggered by the feature of BMAT capable of storing and accumulating lipids as large unilocular lipid droplets (4). This organelle formed during adipogenesis allows for BMAT to be responsible for the storage, mobilization, and metabolism of fatty acids, which are important metabolic substrates for adipose tissue (4, 41). However, in late stages of differentiation, adipocytes also synthesize other adipose tissue-specific products and secreted products (42), which are not included in the GO terms of ‘lipid’ and ‘fatty acid’, leading to the potential bias in search of effective compounds.

One the other hand, we hypothesized that not all of the DE genes directly contribute to a physiological process or a disease. Some of them could represent a “secondary effect” activated by the actual causal genes and do not offer additional information but give a strong noise when used in signature matching. The ideal way is to employ gene signatures, which directly contribute to the phenotypes. However, only using omics data is difficult to identify all of the relevant genes. Therefore, we employed a reduced gene set created by integrating our transcriptomic profiles with gene ontology sources implementing established biological processes and molecular functions related to “lipid” and “fatty acid” to direct target adipogenesis. This method provided us with a unique subset of genes related to important aspects of adipogenesis, and pave the way for exploring compounds targeting adipogenic differentiation.

### Rationale behind selection of time points for CMap analyses

Dynamic gene expression profiling allows us to capture gene sets characterizing the sequential differentiation stages from BMSCs to fully differentiated adipocyte. DE probes at day 4 were selected for the computational compound screening in CMap on the basis of our previous study revealing that BMSCs adopt to committed adipocyte phenotype following 48–96-hour exposure to adipogenic medium, which was reflected by the stabilization of number of DE probes in the early stage of differentiation (day 0 - day 4) (29). This finding is supported by the observation showing that lipid droplets are visible after 3-5 days of adipogenic differentiation, only to accumulate further from then onwards (43). On the other hand, most DE genes were observed around day 14 following adipogenic induction as opposed to earlier (day 7) or later stages (day 21) (44). These studies provided us with evidence to select day 13 as a second important time point for CMap query analysis.

### Identification of two novel compounds that inhibit BMSC-derived adipogenesis

Emetine is a natural alkaloid originally isolated from the plant *Psychotria ipecacuanha* (45). This alkaloid has been used as therapeutic target against multiple diseases including amoebiasis (FDA approved), leukemia, Zikavirus, Ebolavirus, cytomegalovirus and SARS-CoV-2 infection (46–50), and was identified as an inhibitor regulating cell autophagy (46). Autophagy is an essential process for adipogenic differentiation (51). Inhibition of autophagy is of significant importance to alter differentiation state and metabolic function of adipocytes. Knockdown of Autophagy-related 7 (*Atg7*) in 3T3-L1 preadipocytes suppresses lipid accumulation and blocks the expression of adipogenic marker proteins (52), while others have been demonstrated that sustained activation of autophagy suppresses adipocyte differentiation. In our study we found autophagy related genes such as *ATG14*, *ATG7* and *ULK1* to be upregulated following treatment with emetine in adipogenic-BMSCs in a dose-dependent manner (**Figure S4**) indicating the activation of autophagy in cells by emetine. These observations provide mechanistic insight into the involvement of autophagy in emetine-modulated adipogenic differentiation of BMSCs.

The other compound we identified as inhibitory for adipogenic differentiation, kinetin-riboside, was identified as a natural product found in coconut fruits in nanomolar scale (53, 54). It is well established as an anti-tumor molecule able to suppress various kinds of cancer types such as leukemia, colon adenocarcinoma and hepatoma through its anti-proliferative activity and apoptotic effects (55). While its precise mechanism of action is not clear, kinetin-riboside induced apoptosis through classical mitochondria-dependent apoptotic pathways (56). The chemical disrupts mitochondrial membrane potential, induces release of cytochrome C to the cytosol and activates the pro-apoptotic protein caspase 3 (56–59). Mitochondria biosynthesis is well illustrated for its linkage with adipogenesis depending on the type of adipose tissue (59). Studies have shown that proper mitochondrial activity is required for adipogenic differentiation from MSCs and maturation of adipocytes (60, 61). Given the importance of mitochondrial activities in multiple events of adipogenesis, this could explain why kinetin-riboside treatment demonstrated anti-adipogenic effect at all three stages of adipogenesis.

Bone marrow adipocytes have both overlapping and distinct morphology and physiology, along with different transcriptomic profiles compared to adipocytes from white adipose tissue or brown adipose tissue (4, 5). Although our research question related to mesenchymal stromal cell transition to adipocytes does not apply to the other fat depots in our body, we recognize the current limitation of our study of not having included adipocytes from other depots and their response to our newly identified compounds. In fact, future studies should reveal whether the compounds are specifically acting on bone marrow stromal cell-derived adipocytes or whether they also act on adipocytes from other fat depots.

Altogether, we showed that by systemically enriching adipocyte-specific DE probes from transcriptomic data during BMSC-derived adipogenic differentiation, a list of anti-adipogenic compounds was created. All the compounds we tested showed promising anti-adipogenic effects *in vitro*. We identified two new molecules to have inhibitory actions during adipogenic differentiation or adipocyte maturation, making them potentially interesting drugs to oppose excessive adipocyte accumulation. These findings demonstrate that our analytical and experimental pipeline can be used in the drug repurposing field.

## Data availability statement

The original contributions presented in the study are included in the publication and supplementary material; further inquiries can be directed to the corresponding author. All of the microarray data and CMap data that are at the basis of this study have been deposited in Gene Expression Omnibus (GEO) with the accession numbers GSE80614 and GSE70138, respectively.

## Author contributions

SZ, NL, JP, JL and BE designed the studies. SZ and MK performed the experiments and interpreted the data. SZ wrote the manuscript. All authors contributed to the article and approved the submitted version.
