# Supplementary figures and images for: Identification of small molecules as novel anti-adipogenic compounds based on Connectivity Map
Source: Front Endocrinol (Lausanne). 2022 Dec 16;13:1017832. doi: 10.3389/fendo.2022.1017832 (PMC9800878; doi:10.3389/fendo.2022.1017832)

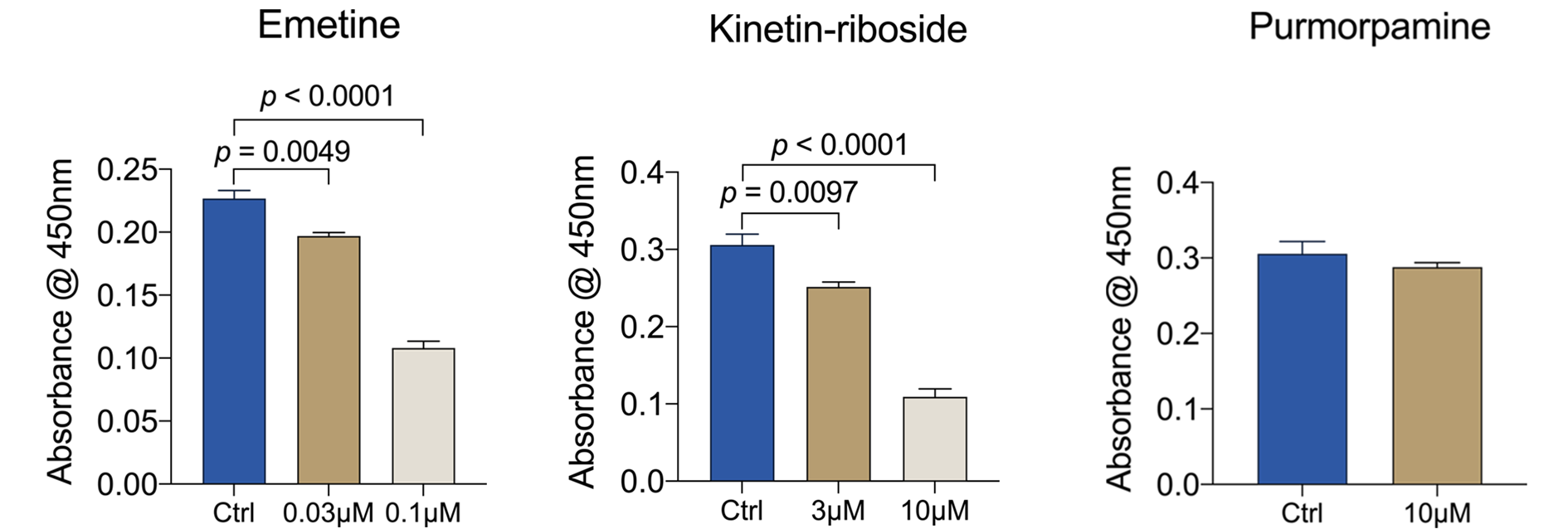

Supplement: Supplementary file 3 [file Image_3.tif]

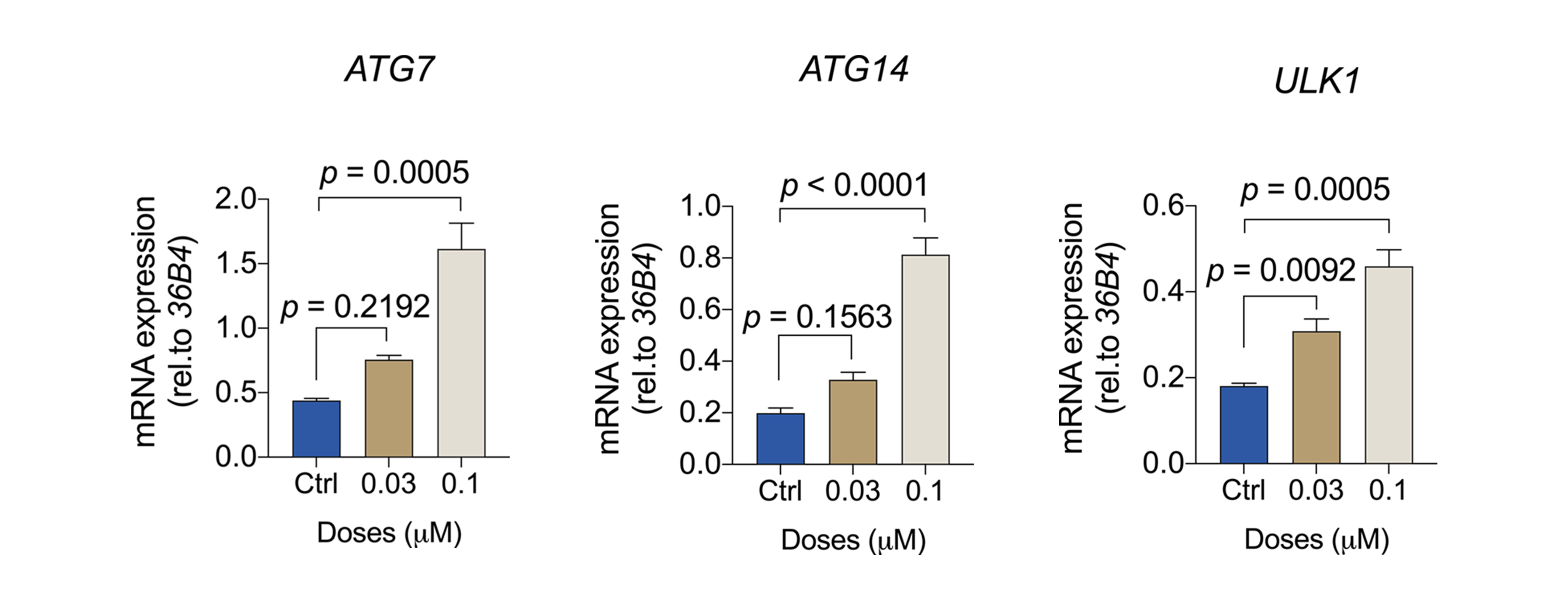

Supplement: Supplementary file 4 [file Image_4.tif]
