# Supplementary material for: Identification of small molecules as novel anti-adipogenic compounds based on Connectivity Map
Source: Front Endocrinol (Lausanne). 2022 Dec 16;13:1017832. doi: 10.3389/fendo.2022.1017832 (PMC9800878; doi:10.3389/fendo.2022.1017832)
Supplement: Supplementary file 5 [file Table_1.docx]

**Table S1 Primer sequences used for qRT-PCR in this study**

| **Name** | **Sequence** |
| --- | --- |
| *FABP4*-forward | TACTGGGCCAGGAATTTGAC |
| *FABP4*-reverse | GGACACCCCCATCTAAGGTT |
| *PLIN1*-forward | CCTGCCTTACATGGCTTGTT |
| *PLIN1*-reverse | ATTCTCCTGCTCAGGGAGGT |
| *ADIPOQ*-forward | AACATGCCCATTCGCTTTACC |
| *ADIPOQ*-reverse | TAGGCAAAGTAGTACAGCCCA |
| *36B4*-forward | GTCCTCGTGGAAGGCCC |
| *36B4*-reverse | AGGAGAGACAGGGAGCTCAG |
